# 3D-printed guides for cervical pedicle screw placement in primary spine tumor: Case report and technical description

**DOI:** 10.3389/fsurg.2022.1011846

**Published:** 2022-11-23

**Authors:** Nicola Marengo, Giuseppe Di Perna, Bianca Maria Baldassarre, Fabio Cofano, Raffaele De Marco, Pietro Zeppa, Salvatore Petrone, Marco Ajello, Diego Garbossa, Francesco Zenga

**Affiliations:** ^1^ Department of Neuroscience “Rita Levi Montalcini,” Neurosurgery Unit, University of Turin, Turin, Italy; ^2^ Skull Base and Pituitary Surgery Unit, AOU Città Della Salute e Della Scienza, Turin, Italy; ^3^ Spine Surgery Unit, Casa di Cura Clinica Città di Bra, Bra, Italy; ^4^ Spine Surgery Unit, Humanitas Gradenigo Hospital, Turin, Italy

**Keywords:** craniovertebral junction (CVJ), cervical pedicle screw (CPS), chordoma, spine tumor, 3D-printed guides, case report

## Abstract

**Introduction:**

For spine surgeons, dealing with unstable cervical spine has been usually challenging, and this becomes more difficult when facing a primary craniovertebral junction tumor. Primary spine tumor surgery should always include column reconstruction in order to guarantee biomechanical stability of the spine, but surgeons should always be aware that instrumentations could create interferences with postoperative radiations. However, although carbon fiber instrumentations have started to be used in thoracolumbar oncology for few years, these options are still not available for cervical spine. In the reported case, the adopted strategy to obtain adequate column reconstruction was based on the idea of reducing the amount of titanium needed for posterior fixation and maximizing the distance between the radiation target and titanium rods.

**Case report and aim:**

We present the case of a 53-year-old woman harboring a craniovertebral junction chordoma. A short occipito-C3 construct was selected. Specifically, titanium cervical pedicle screws were placed by using a new technology consisting in patient-tailored and customized 3D-printed guides. The aim of this case report is to determine the feasibility and safety of 3D-printed guides for cervical pedicle screw (CPS) positioning, even in the case of cervical spine tumor.

**Conclusion:**

CPS could represent a good solution by providing strong biomechanical purchase and tailored 3D-printed guides could increase the safety and the accuracy of this challenging screw placement, even in oncological patients.

## Introduction

For spine surgeons, dealing with unstable cervical spine has been usually challenging, especially when facing a primary craniovertebral junction (CVJ) tumor. Although the best management of CVJ chordomas is still a matter of debate, the majority of studies showed that the extent of a resection was associated with the best progression-free survival and overall survival rate (1, 2). Moreover, because of the neurovascular structure crowding and the consequent frequent impossibility of performing an en-bloc removal of these tumors, the surgery goal should aim at maximal safe removal providing a safe target for subsequent proton-beam radiation therapy (3–6).

However, surgical considerations should always include column reconstruction in order to guarantee biomechanical stability of the spine, being aware that column reconstruction should also consider interferences between radiations and instrumentations, with consequent lowering of effective radiation dose on the target (7–10). During the last few years, carbon fiber/PEEK implants have been routinely used in thoracolumbar spine reconstruction being able to reduce artifacts and scattering effects while maintaining adequate mechanical properties (11, 12). However, these implants are not fully available for cervical spine reconstruction; thus, other solutions should be adopted.

In the reported case, the surgical strategy sought to obtain adequate column reconstruction while reducing the amount of titanium for posterior fixation and maximizing the distance between the radiation target and titanium rods.

Indeed, a short occipito-C3 construct using titanium cervical pedicle screws (CPS) was used. CPS placement could be really challenging: the misplacement rate ranged from 6% to 30% with different techniques. Although the radiological misplacement does not necessarily give clinical consequences, as described by Mahesh et al. (13), cases with neurovascular complications caused by CPS insertion were described in the literature (14, 15).

Therefore, in the presented case, a new technology of patient-tailored and customized 3D-printed guides was used to fix CVJ of a 53-year-old patient harboring a chordoma.

The aim of this case report is to determine the feasibility and safety of 3D-printed guides for CPS positioning even in the case of cervical spine tumor.

## Case description

**Figure 1:**
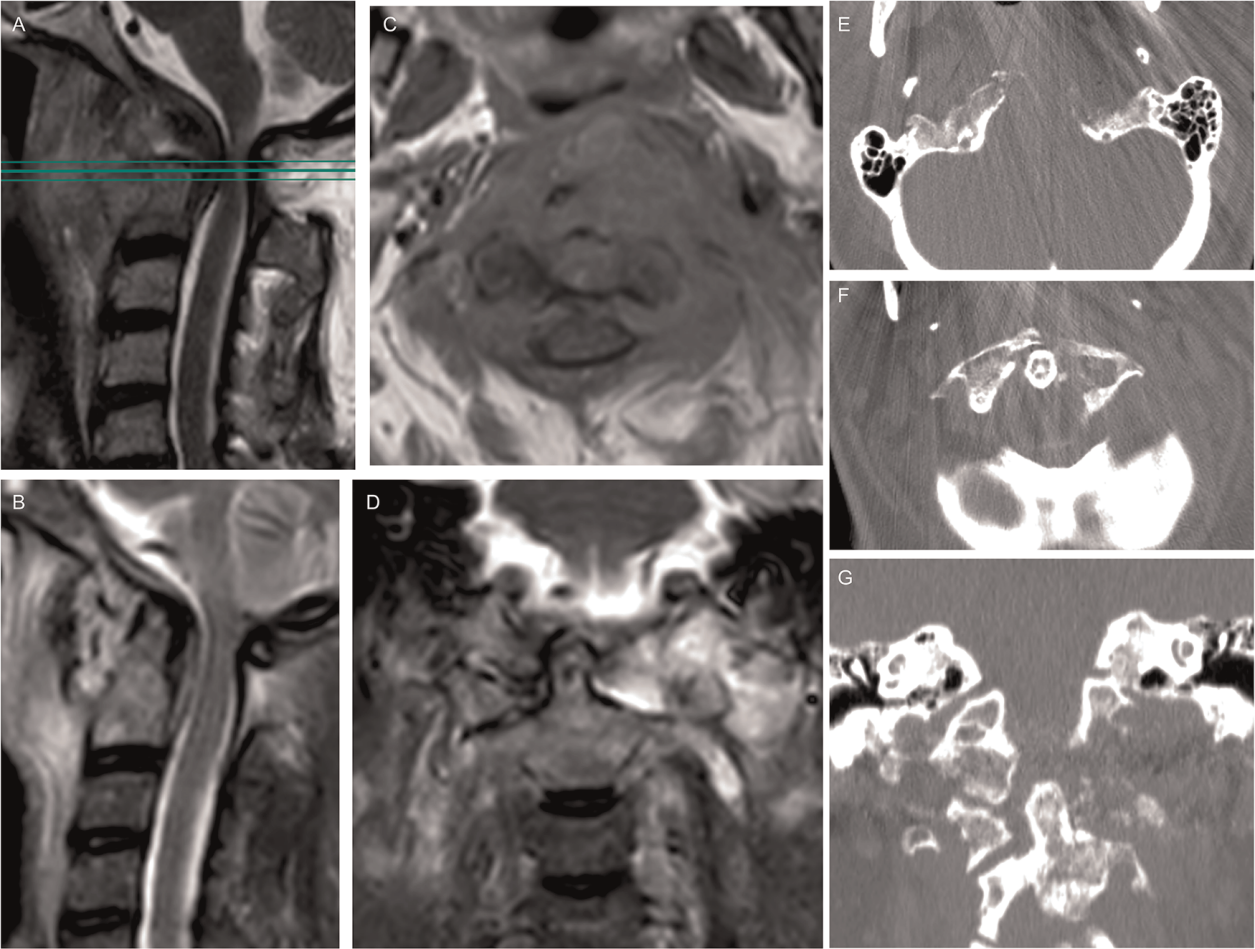
Preoperative spine MRI showed a compression of the midbrain at the craniovertebral junction on the STIR sequence (**A**); an axial section contrastenhanced T1-weighted (**C**) depicted a low-enhancing lesion with extension from the clivus, left petroclival fissure, and left condyle up to the prevertebral plane of the fifth cervical vertebral body, determining an anterior dislocation of the oropharynx and an osteolysis of the left part of anterior C1 arch; the lesion showed an hyperintensity in T2-weighted images (**B, D**). Preoperative CT scan showed an osteolytic involvement of the left condyle and lateral mass (**E-G**) causing instability of the craniovertebral junction.

A 53-year-old female patient with 6-month history of neck pain and dysphagia was presented to the authors' attention for an expanding left cervical mass. No neurological symptoms were reported, and the patient was also affected by hypertension, diabetes, and ischemic cardiopathy. Contrast-enhanced magnetic resonance imaging (MRI) and computed tomography (CT) scans showed an extensive skull base tumor, located in the left paravertebral space, growing from the clivus and reaching the left anterior portion of C1 arch, the left occipital condyle compromising both C0–C1 and C1–C2 left joints (Figure 1). A needle CT-guided biopsy was performed with histological confirmation of chordoma.

Due to extensive bone involvement, a single-day two-steps surgery was planned. First, the tumor involving the clivus and the anterior C1 arch was approached anteriorly through a pure endoscopic endonasal route then a posterior “open” approach was performed to complete resection of tumor involving the left condyle and to fix CVJ by using a C0–C3 construct.

**Figure 2:**
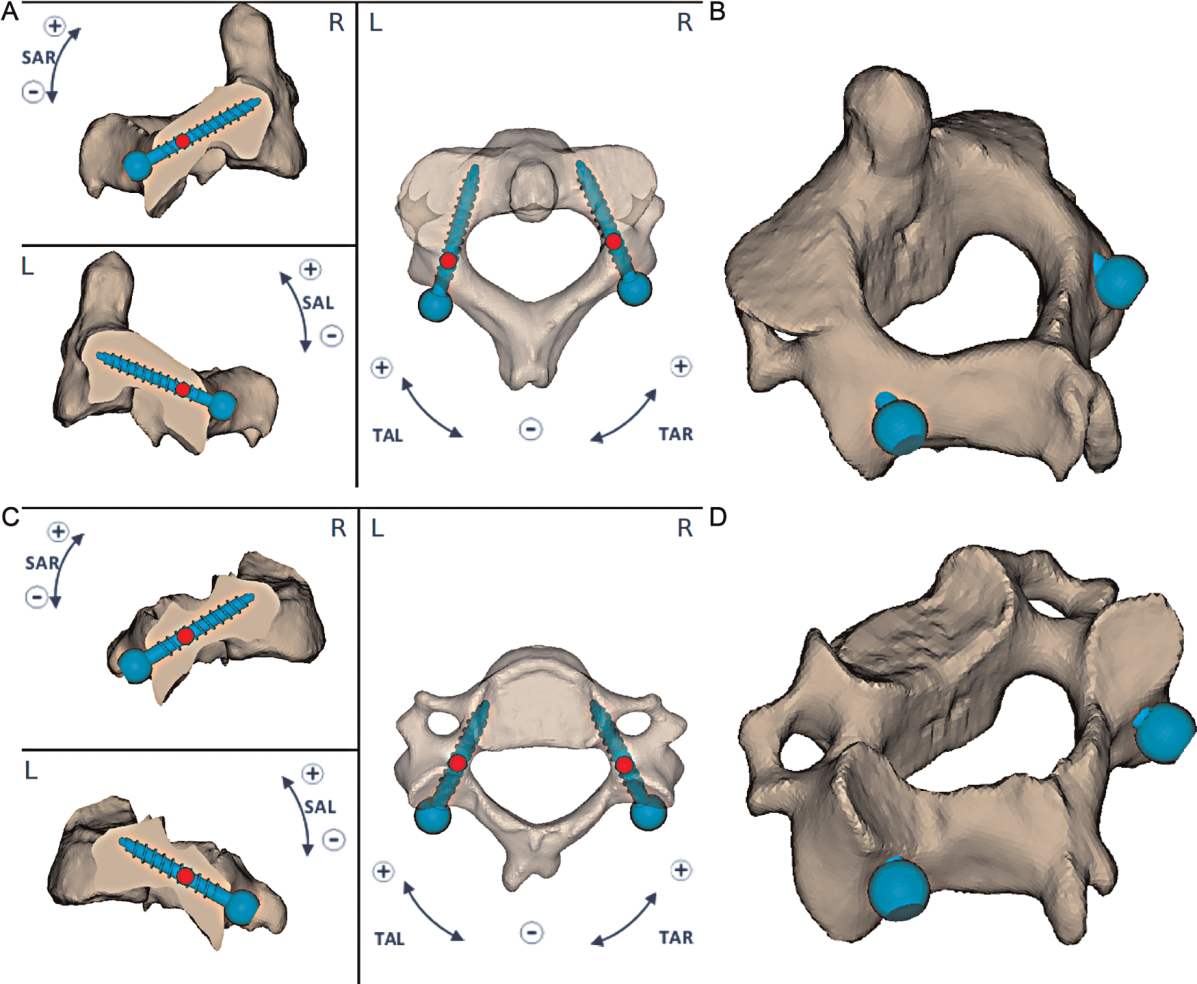
An occipitocervical fusion was planned after endoscopic endonasal removal of the lesion. A preoperative planning of C2–C3 pedicle screws was reproduced. The 3D-printed guides were manufactured considering values of sagittal plane angle (SAL or SAR) and transverse plane angle (TAL or TAR) of screws, horizontal and vertical distances, screw directions and angulations and their entry points (**A**, **C**); C2 and C3 3D planning were shown (**B**, **D**).

A short construct was planned due to the patient's comorbidity; thus, individualized 3D-printed guides (MySpine Cervical, Medacta, Rancate, Switzerland) were developed for the placement of C2 and C3 pedicle screws in order to increase the construct purchase and to guarantee the safety of surgery (Figure 2).

### Surgical technique

#### Endoscopic endonasal step

The procedure was performed by using total intravenous anesthesia to allow intraoperative neuromonitoring (IONM). Once reached the lower clivus and the CVJ by using expanded endoscopic endonasal approach—as already reported in previous papers by the senior author (16–19)—a huge mass was encountered into the rhinopharynx, and there was no chance to harvest the usually reverse *U*-shape flap. Therefore, a right naso-septal flap (NSF) was harvested with a laser. After checking the tumor's margin with neuronavigation system, partial circumferential dissection was started. The extreme lateral part of the tumor was tightly attached to the pharyngeal muscles; thus, only internal tumor debulking was carried out followed by centripetal dissection of tumor margins. Then, once the involved part of anterior C1 arch was removed, a left condylectomy was performed through a far medial extension of the approach completing tumor removal planned from the anterior. No dural tears and/or CSF leaks were encountered at the end of this step. The osteo-dural defect was covered by the harvested NSF and secured with fibrin glue.

#### Posterior removal and CVJ fixation

**Figure 3:**
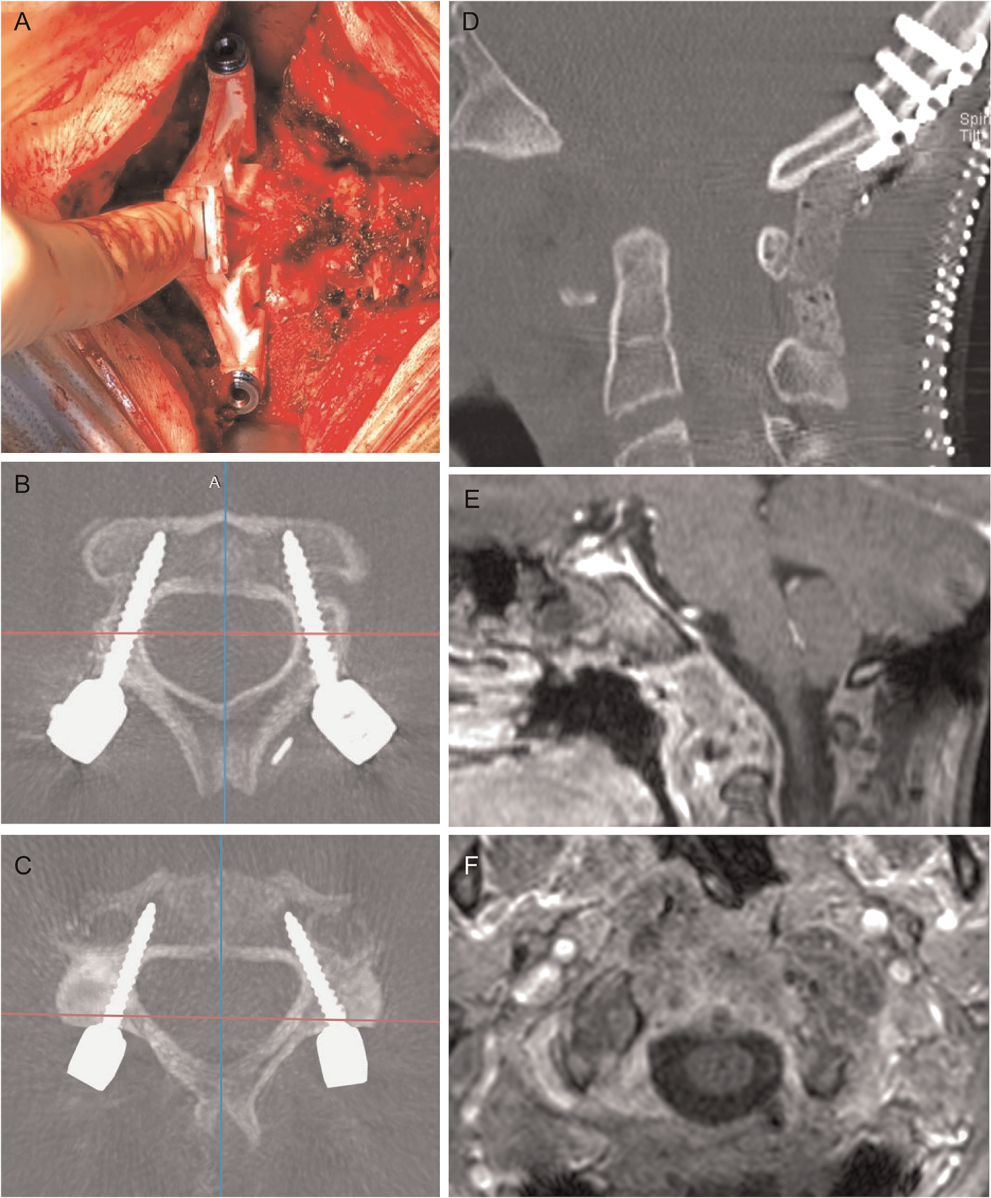
Intraoperative view of the printed guide fitting on C3 lamina (**A**) and postoperative results. Transversal CT scan of screws' trajectories in C2 and C3, respectively (**B**, **C**). The result of anterior decompression was shown in (**D**) and an occipital plate and bicortical screws connected with precontoured rods completed the occipitocervical constructs. One month brain and spine MRI (**E**, **F**) confirms the decompression of the cervicomedullary junction and the optimal extent of resection.

The patient was placed in the prone position, and her head was fixed with a three-pin head holder. IONM remained stable during patient re-positioning. A midline skin incision from the inion to C3 spinous process was performed. Subperiosteal dissection exposed the occiput, C1, C2, and C3 posterior elements. A rigid plate was fixed to the occiput by using bicortical screws. Due to the significant C1 involvement and extended tumor removal, C1 was not included in the fusion. Once the anatomical landmarks on C2 and C3 posterior surface were exposed by meticulous soft tissue removal, the individualized-3D-printed guide was docked first on the C2 vertebra, and pedicle screws were inserted. Then with the same technique, C3 pedicle screws were positioned. Finally, a pair of precontoured rods was used for stabilization. Cancellous bone was laid on the midline between occiput and the C2 to promote fusion (Figure 3D). The posterior decompression was performed after screw positioning. C1 posterior arch was removed along with the left lateral mass, through which was possible to reach the margin of anterior resection (Figure 3).

Postoperative course was uneventful. Postoperative cranio-cervical CT scan and 1-month MRI assessed the accuracy of screw placement and confirmed the gross total intralesional removal.

Patient neurological status was absolutely preserved at discharge. Histological exam confirmed the diagnosis of low differentiated chordoma and patient was scheduled for proton-beam radiation therapy.

### 3D-printed guide features

The guides are custom-made devices intended to be used as anatomical perforating guides, specific to a single patient's anatomy, to assist intraoperatively in the positioning of screws during posterior cervical fixation surgery. A preoperative surgical planning software (MySpine Surgical Planning Report, Medacta, Rancate, Switzerland) utilizes patient's thin section spine CT scan to plan the best screw trajectory, its entry point, and dimensions in order to reach the best achievable biomechanical features. A drill-based technique, with Stryker system dual-trigger rotary handpiece (Stryker Corporation, Kalamazooo, MI, USA) was used to make a guiding hole anticipating the final screw placement.

The preoperative planning assesses the main surgical parameters regarding the screw implantation in order to manufacture a dedicated single patient-matched 3D-printed guide. Any modifications of the preoperative planning are defined by an exclusive confrontation between the surgeon and manufacturer. The surgeon is able to choose the guide configuration and modify surgical parameters considering screws' diameters, lengths, and angulations in relation to the sagittal, coronal, and axial planes. Moreover, when planning surgery, a surgeon should carefully ensure that the screw axis was placed completely inside the cancellous bone due to the strict range of acceptable accuracy error for CPS.

The 3D-printed guides are defined by two main features, providing maximum stability: one central contact (1) and two lateral cylindrical guides (left and right) (2) with distal pins. These points of contact with spinous process, laminae, and lateral masses are useful to optimize the stability of the guide with the specific vertebra reproducing accordingly the planned screw entry points.

Furthermore, depending on the patient's matched anatomic model, the guide can be customized to maximize the contact to one of the following areas: spinous process and laminae, spinous process and lateral masses, and laminae and lateral masses. According to the spinous process conformation, the posterior profile of the guide could be printed in three different shapes being available open, semi-open, and closed configurations.

Lastly, another 3D model of the interested vertebra was available in the operative room, allowing surgeons to confirm the insertion point and fitting conditions of the 3D-printed guide with the vertebra by easily comparing the intraoperative spine and the 3D model each time before and after probing and tapping.

## Discussion

The best management of spinal chordomas is still a matter of debate, and identifying the best surgical and oncological approaches represents the real challenge (1). Di Maio et al. reported a meta-analysis of 23 retrospective studies showing that the gross total resection was associated with the best progression free survival and overall survival rate (20). Nonetheless, because of the impossibility of en-bloc resection due to the crowding of neurovascular structures, intralesional removal is the treatment of choice for CVJ chordomas (1, 20–26). Surgery should aim to obtain maximal safe resection providing a safe target for subsequent focused radiation therapy, being this strategy closer to the treatment of metastatic spine tumors (27, 28).

Nowadays, particle therapy, including proton therapy, has shown unique physical properties, being able to spare normal tissues from unnecessary exposure while providing high radiation doses to the target (3, 5). Hence, early postoperative proton beam therapy represents the radiation treatment of choice for chordomas, allowing them to deliver high radiation doses while sparing adjacent neural structures (4, 5, 29).

However, when dealing with spine tumors, surgical considerations should always include column reconstruction in order to guarantee biomechanical stability of the spine (30). Specifically, in the case of a primary spine tumor that is a priori unresectable with wide margins and that would certainly need proton beam therapy, column reconstruction should not only aim to provide stability, but should also consider interferences between radiations and instrumentations.

Indeed, many manufactured metals used for prostheses could absorb radiation, decreasing radiotherapy effectiveness (7–10). Carbon fiber/PEEK implants have been developed and used in spinal oncology, showing encouraging results in reducing artifacts and scattering effects, while maintaining adequate mechanical properties (11, 12). However, while carbon fiber/PEEK instrumentation is fully available for thoracolumbar spine, there is a lack of radiations inert materials for cervical spine instrumentations.

In the reported case, the adopted strategy to obtain adequate column reconstruction was based on the idea of reducing the amount of titanium needed for posterior fixation and maximizing the distance between the radiation target and titanium rods.

Therefore, CPSs were used for occipitocervical fixation of C2 and C3 vertebras, allowing for a short construct due to the strong purchase provided by pedicle screws. Moreover, the titanium rods were preoperatively contoured in a laterally convex fashion in order to maximize the distance of titanium from the tumor site, aiming to reduce the radiations scatter effect. Another described option—considered in the preoperative planning—was those reported by Boriani et al., who used a hybrid solution combining titanium sublaminar bands and carbon fiber/PEEK rods (31). However, CPSs were preferred to sublaminar bands to obtain a stronger fixation of the CVJ. Analyzing the biomechanics of five occipitocervical constructs, Oda et al. showed a higher stability provided by occipital screw and CPSs connected by occipitocervical rods (32). Furthermore, a correction of CVJ kyphosis could be obtained by adequate rods contouring and by applying a distraction force between the occipital screws and CPSs (33).

As widely reported in the literature, although the use of CPSs has shown to provide stronger fixation than alternative methods—that is, lateral mass screws and trans-articular facet screws—due to the biomechanical superiority of CPSs, CPSs placement is associated with considerable risks of nerves or vertebral artery injuries (14, 34, 35). Indeed, a screw perforation rate ranging from 6.7% to 29% has been reported using the free-hand screw insertion technique that seems to be lowered by using intraoperative image devices (36–38), such as fluoroscopy-guided technique (14, 39), 3D fluoroscopy (40), CT-based navigation system (41, 42), and O-arm (43, 44). Recently, the use of 3D-printed guides has been proposed as a new technique for CPSs placement.

There are few experiences in the literature describing the use of these guides but they seem to be encouraging (45–49). Kaneyama et al. reported a high rate of accuracy (97.5%) in a series of 80 screws placed with 3D-printed templates (48). Fujita et al. recently reported their experience with the same 3D-printed guides adopted in this case report, showing interesting and promising results with 98.7% of accuracy (49).

Two main issues have been advanced for the accuracy of CPS placement because of a higher mobility of cervical vertebra compared with lumbar or thoracic ones, and fewer anatomical landmarks on the posterior surface of cervical vertebra (43, 50).

The relevant mobility of cervical spine could lead to vertebra rotation during screws placement maneuvers (drilling, probing, and tapping) with consequent changes in cervical alignment, thus increasing the risk for screw misplacement due to the different tapping and screw trajectories. Moreover, the few landmarks on the posterior aspect of cervical vertebra and the wide anatomical variations in size and shape of cervical pedicles contribute to increase the risk of screws misplacement (51, 52). Cervical pedicles, indeed, could be really small making the screw positioning challenging with free-hand and/or fluoroscopy assisted techniques.

The smallest mean pedicle width was about 4.5 mm and was usually observed at C3. A gradual increase in the mean value was observed from C3 to C7 (38). Liu et al. reported racial and sexual differences in pedicles' diameters, being smaller in Asians than in Europeans or Americans and among female individuals of both races than their male counterparts (51, 53).

Lastly, another factor that seems to increase the risk of misplacement is the soft tissue of the neck, namely, muscles and fat tissue, that could contribute to a muscle-pushing effect, which can lead to screw malpositioning. Apparently, it could impact more on screw misplacement than the pedicle diameter (38).

Patient-specific 3D-printed guide resulted to be very useful in the presented case. Surgeons were able to replicate the preoperatively planned screw trajectory as the 3D-guides were fixed to each target vertebra. The guide, indeed, provided a highly accurate copy of the planned entry points and trajectories. Moreover, due to the availability of a 3D-printed guide for each vertebra, the effect of intraoperative spine alignment change was completely overcome. This is strictly related to the 3D-guide purchase on the interested vertebra and it was provided by some specific features. The former was the presence of the caudal hook on the 3D-printed guide - inherited from the thoracolumbar system (Medacta, My Spine MC) – which strongly held the guide to the caudal aspect of the vertebra, stabilizing the complex on a sagittal plan; the latter was the availability of the 3D model in the operative room, allowing real-time confirmation of the planned landmarks and the fitting conditions of the 3D-printed guide with the vertebra before and after probing and tapping. As underlined, the guide fitting is essential to guarantee screws placement accuracy, then some cautions should be emphasized. First, as guides are printed on the basis of preoperative bone CT, accurate reproduction of bone structures, namely the shape of the lamina and lateral mass surface, could be thwarted by the presence of cartilaginous tissues or osteophytes, especially in severe degenerative pathology. Since this could lead to screws misplacement, surgeon should carefully remove soft tissue and osteophytes on the posterior aspect of the vertebra where the guide needs to be positioned. Moreover, paraspinal muscles should also be retracted to adequately engage the 3D-guide. Notably, as the CPS entry point becomes lateral in the cranially located vertebras, the distance between the lateral cylindrical elements of the 3D-printed guide and so the entire axial dimension of the guide will become wider. Therefore, it is important to make sufficient skin incision, especially on the cranial side, in order to achieve adequate guide fitting.

Although the reported experience has several limitations, starting from its nature of case report, the result of accuracy in CPS placement seems to be encouraging and it could be a starting point for further investigation even in the oncological field. Another limitation is represented by time which is required for manufacturing and obtaining an adequate preoperative planning (about 2 or 3 weeks). However, due to the complexity of oncological patients and because the majority of procedures are elective, this usually does not result in treatment delay.

## Conclusions

The treatment of unstable cervical spine remains challenging, especially when dealing with primary spine tumor. Despite oncological principles of radicality, CVJ chordoma represents an en-bloc unresectable tumor, and therefore, postoperative proton beam therapy plays a crucial role in controlling progression free survival. Hence, every effort should be carried out during surgery in order to guarantee a safe and clean target for radiation. Short titanium constructs could consider whether carbon fiber instrumentation could not be used or could not provide adequate stability. CPS could represent a good solution by providing strong biomechanical purchase, and tailored 3D-printed guides could increase the safety and accuracy of this challenging screw placement, even in oncological patients.

## Data Availability

The raw data supporting the conclusions of this article will be made available by the authors, without undue reservation.
